# COVID-19 vaccine hesitancy among the adult population in Ghana: evidence from a pre-vaccination rollout survey

**DOI:** 10.1186/s41182-021-00357-5

**Published:** 2021-12-16

**Authors:** Robert Kaba Alhassan, Matilda Aberese-Ako, Phidelia Theresa Doegah, Mustapha Immurana, Maxwel Ayindenaba Dalaba, Alfred Kwesi Manyeh, Desmond Klu, Evelyn Acquah, Evelyn Korkor Ansah, Margaret Gyapong

**Affiliations:** 1 Institute of Health Research, University of Health and Allied Sciences, PMB 31 Ho, Ghana; 2 Centre for Health Policy and Implementation Research, Institute of Health Research, University of Health and Allied Sciences, PMB 31 Ho, Ghana

**Keywords:** Coronavirus disease 2019, Vaccine, Immunization, COVID-19, Trial, Willingness, Uptake, Ghana

## Abstract

**Background:**

Coronavirus disease 2019 (COVID-19) has already claimed over four million lives globally and over 800 deaths in Ghana. The COVID-19 vaccine is a key intervention towards containing the pandemic. Over three billion doses of the vaccine have already been administered globally and over 800,000 doses administered in Ghana, representing less than 5% vaccination coverage. Fear, uncertainty, conspiracy theories and safety concerns remain important threats to, a successful rollout of the vaccine if not managed well.

**Objective:**

Ascertain the predictors of citizens’ probability of participating in a COVID-19 vaccine trial and subsequently accept the vaccine when given the opportunity.

**Methodology:**

The study was an online nation-wide survey among community members (*n* = 1556) from 18th September to 23rd October, 2020 in the 16 regions in Ghana. Binary probit regression analysis with marginal effect estimations was employed to ascertain the predictors of community members’ willingness to participate in a COVID-19 vaccine trial and uptake the vaccine.

**Results:**

Approximately 60% of respondents said they will not participate in a COVID-19 vaccine trial; 65% will take the vaccine, while 69% will recommend it to others. Willingness to voluntarily participate in COVID-19 vaccine trial, uptake the vaccine and advise others to do same was higher among adults aged 18–48 years, the unmarried and males (p < 0.05). Significant predictors of unwillingness to participate in the COVID-19 vaccine trial and uptake of the vaccine are: married persons, females, Muslims, older persons, residents of less urbanised regions and persons with lower or no formal education (*p* < 0.05). Predominant reasons cited for unwillingness to participate in a COVID-19 vaccine trial and take the vaccine included fear, safety concerns, lack of trust in state institutions, uncertainty, political connotations, spiritual and religious beliefs.

**Conclusion:**

The probability of accepting COVID-19 vaccine among the adult population in Ghana is high but the country should not get complacent because fear, safety and mistrust are important concerns that have the potential to entrench vaccine hesitancy. COVID-19 vaccine rollout campaigns should be targeted and cognisant of the key predictors of citizens’ perceptions of the vaccine. These lessons when considered will promote Ghana’s efforts towards vaccinating at least 20 million people to attain herd immunity.

**Supplementary Information:**

The online version contains supplementary material available at 10.1186/s41182-021-00357-5.

## Background

Humanity has been confronted with one form of pandemic or the other, dating back to the Black Death pandemic (1347–1351) which killed over 200million people [1] to the current Coronavirus disease 2019 (COVID-19) pandemic. COVID-19  has so far infected over 180 million people and killed more than four (4) million victims globally as at July, 2021 [2]. Ghana’s experience of pandemics has been particularly terrible from the Russian Flu of 1889–1891 to the Spanish Flu of 1918–1919 when an estimated 60,000 people died [1]. In Ghana, COVID-19 has infected over y 100,000 people and killed over 800 deaths as at July, 2021 [3, 4].

Vaccines are considered as means of protection. Thus, COVID-19 vaccines have been introduced as a means to curtail transmission for the world to transit into post-COVID-19. However, this has been met with hesitancy, fear and safety concerns coupled with anti-vaccine conspiracy theories which significantly compromise acceptance and willingness to accept the vaccine. Lack of adequate information on the principles behind vaccine development from phases one, two and three trials which take into account, immunogenicity, safety and efficacy issues, by the average citizen partly account for the high level of fear, anxiety and uncertainty associated with the vaccine acceptance and uptake. Moreover, the all-time record development of the COVID-19 vaccine particularly has been cited as a key factor in the hesitancy associated with the vaccine uptake in many parts of the world, including Ghana hence the need for this study.

According to the World Health Organization (WHO), as of 13th July 2021, 3 billion vaccine doses had been administered globally representing 25% of the world’s population receiving at least one dose; barely 1% of persons living in low-income countries have received at least one dose [5]. There are at least 7 different vaccines on three platforms approved by the World Health Organization (WHO) [4]. Within the West African sub-region, Ghana was the first country to take delivery of the first consignment of COVAX facility of 600,000 Oxford AstraZeneca vaccine on 24th February, 2021 [5]. The vaccine was subsequently deployed on 2nd March, 2021 with the President, senior political personalities, traditional and religious leaders taking the vaccination shots publicly to engender public trust and confidence [6].

Over 800,000 doses of the COVID-19 vaccines were administered in Ghana as at May, 2021. However, at the time of writing this paper, there was no available publication on the side effects experienced by persons who took the AstraZeneca vaccine and how these experiences potentially correlate with likelihood of accepting a second jab or recommending it to others. Even though anecdotal data suggests some isolated cases of reported adverse events after taking the vaccine, these reports are not yet reported in a consolidated manner supported by scientific data.

An estimated 20 million Ghanaian citizens out of the over 30 million Ghanaians were targeted to receive the first batch of the vaccine with priority given to hot spot regions (i.e. Greater Accra, Ashanti and part of the Central regions) in the country. In addition, priority was given to frontline health workers, persons aged 60 and above and those with underlying medical conditions. During the deployment, an additional 50,000 AstraZeneca vaccines were donated by the Indian government to Ghana on 5th March, 2021 [7]. To promote uptake of the vaccine, the Ghana government adopted multi-faceted health education approaches, such as use of print, electronic and social media. These communications strategies were led by the ministries of information and communication, information services department, ministry of health and Ghana Health Service (GHS). Messages were further communicated to the grassroots using local community radio and television stations in the local dialects. The role of civil society organizations and non-governmental organizations (NGOs) on health was also phenomenal.

Like many health interventions. vaccinations generally come with risks and benefits which need to be acknowledged and duly communicated to potential beneficiaries of these interventions. As part of efforts to promote safety and reduce risks, vaccines undergo rigorous stages (phases 1, 2 and 3) of development, where data safety monitoring boards (DSMBs) play a critical role to guaranteeing patient safety. With particular reference to the COVID-19 vaccines, available literature shows the vaccines reduce morbidity and mortalities in countries with high acceptability rate such as United States of America [8–12], China [13, 14], Kuwait [15], Democratic Republic of Congo [16] and some African countries [17]. Nonetheless, fear, anxiety, misinformation, conspiracy theories, lack of trust and confidence in vaccines and government institutions have the potential to impede successful rollout and uptake of the vaccine in many countries across the globe. Consequently, it is important to investigate and understand the socio-economic factors that predict vaccine hesitancy among adult populations in sub-Saharan Africa, particularly Ghana. This study also adopts a sub-group analysis to appreciate the dynamics in vaccine acceptance among the study population to inform targeted messaging to these groups on benefits of vaccines, particularly the COVID-19 vaccine which has proven to reduce hospitalisation and mortalities related to COVID-19.

There is currently no known nation-wide study, at the time of writing this paper, on COVID-19 vaccine hesitancy including willingness to voluntarily participate in a COVID-19 vaccine trial when given the opportunity. This pre-vaccination rollout survey was conducted six clear months before the first batch of COVID-19 vaccines arrived in Ghana for deployment.

## Methods

### Study design

The study was a cross-sectional online survey administered via REDCap software to determine the perceptions on and willingness to participate in COVID-19 vaccine trial and uptake the vaccine in Ghana.

### Study setting and population

The study was conducted across all sixteen (16) administrative regions in Ghana. The target population was all adults aged 18 years and above living in Ghana at the time of conducting the study. Using Krejcie and Morgan’s [18] formula for calculating sample size based on known population, a target sample of 1500 was deemed adequate at 95% confidence level based on the current population of Ghana which is approximately 30 million people.

### Sampling procedure

The sampling procedure was mainly convenience since persons who voluntarily consented to participate and were readily available via the various social media platforms were contacted to answer the questions.

### Inclusion and exclusion criteria

Inclusion criteria for the study participants were persons aged 18 years and above, since they could grant informed consent as adults. In addition, the person should have been resident in the pertinent region at the time of answering the questionnaire to reflect the regional dynamics in response to the COVID-19 vaccine and perspectives on the vaccine trial. In terms of the exclusion criteria, minors and non-residents of the pertinent regions were exempted from the study.

### Instruments of data collection

The study employed an online survey using REDCap software. Link for the questionnaire was circulated via social media platforms and networks between 18th September and 23rd October, 2020. The link for community members was sent to all eligible respondents aged 18 years and above. Respondents who were illiterate or were literate but did not have smartphones to access the electronic tool were assisted to answer the questions by trained community health nurses who were trained as research assistants. Where there was need for physical follow-ups, all COVID-19 protocols were strictly followed, such as wearing of facemasks, handing washing and use of alcohol-based hand sanitizers.

### Structure of data collection tool

The data collection instrument comprised of 60 open and close-ended questions categorized into six (6) main sections. The sections are: Section A (Background information/socio-demographic); Section B (Knowledge and awareness of COVID-19); Section C (Stigma and uptake of COVID-19 testing services); Section D (Adherence to COVID-19 prevention measures); Section E (Impact of the COVID-19 on livelihood) and Section F (Satisfaction with government response to COVID-19). Questions under sections A, B and C were mostly closed-ended (Yes, No) and a few were open-ended (Please specify), while questions under Sections D, E and F were rated on a five-point Likert scale.

The Likert scale for Section D questions ranged from 1 to 5, where 1 means “very rarely”, 2 means “rarely”, 3 means “undecided”, 4 means “most times”, 5 means “all the time”. Section E questions ranged from 1 to 5, where 1 means “very high negative impact”, 2 means “high negative impact”, 3 means “undecided”, 4 means “no negative impact”, 5 means “no negative impact at all”. Finally, Section F Likert scale questions ranged from 1 to 5, where 1 means “very dissatisfied”, 2 means “dissatisfied”, 3 means “undecided”, 4 means “satisfied”, 5 means “very satisfied”.

Internal validity and reliability of the questionnaire items were tested using Cronbach’s alpha test and the average scale reliability coefficient was above the 80% rule of thumb. In addition, the questionnaire was pilot tested to promote internal validity of the test items.

### Recruitment and training of research assistants/data collection

The blended physical and virtual (hybrid) data collection strategy was designed to control self-selection of only persons who could access the online questionnaire on their own. A total of 180 research assistants were trained virtually via Zoom and later assigned to the 16 sixteen regions of Ghana for the data collection. The training on the data collection tool lasted for five (5) days including the day for piloting among themselves virtually due to the COVID-19 restrictions. Follow-up communication with the research assistants was done through phone calls to take stock of the daily number of persons interviewed per research assistants. In addition, daily tracking sheet was filled per research assistants indicating number of persons interviewed, location of interview, mode of interview, outcome of interview and challenges encountered.

### Ethical considerations

Ethical clearance was sought from the Research Ethics Committee (REC) of the University of Health and Allied Sciences (UHAS), Ghana (clearance number: UHAS-REC A.1[6] 20-21) Participation was mainly voluntary and only participants who provided voluntary informed consent were included in the study. Since the survey was administered online and mostly through social media platforms, informed consent was integrated into the online tool, where respondents needed to first consent (Yes, agree) or opt out (No, disagree) to participate in the study. Participants who ticked No or Disagree were automatically denied access to the online questions.

Information from respondents was treated confidentially in accordance with the UHAS-REC guidelines for conducting research throughout the various stages of the evaluation. Given the sensitive nature of the topic, the needed assurance was given to participants that all information collected from participants will be used mainly for this study and will not be made available to a third party without their prior consent.

### Data analysis

Data was analyzed with STATA statistical analysis software (version 14.0) after cleaning and coding for anonymity. Descriptive analysis was conducted for the background socio-demographic variables using frequencies and percentages for the categorical data (gender, educational status, occupation, region of residence, marital status, and religion). Means and standard deviations were used for continuous variables such as age and amount participants are willing to pay for COVID-19 vaccine.

Inferential statistical analysis was done using the binary Probit regression models. The main outcome variables of interest were: (1) COVID-19 vaccine trial hesitancy (defined as probability of respondents participating in a COVID-19 vaccine trial, Yes = 1; No = 0), and (2) COVID-19 vaccine hesitancy (defined as the probability of participants accepting immunization with COVID-19 vaccine ,Yes = 1; No = 0). Explanatory variables of interest were: sex (male = 1; female = 2); age (numerical value); education (categorical multiple response); region (categorical multiple response); marital status (categorical multiple response); religion (categorical multiple response); willingness to pay for COVID-19 vaccine (Yes = 1; No = 0) and knowledge on COVID-19 (Yes = 1; No = 0). All explanatory variables of interest were tested for multicollinearity before fitting them into the regression models. Variables with Variance Inflation Factor (VIF) above the 10.0 rule of thumb were dropped. Variables were few observations were recoded to control multicollinearity. Apart from age, all the remaining explanatory variables were treated as dummy variables because of their categorical nature.

Binary Probit regression analysis using Average Marginal Effect (AME) estimations were employed to ascertain the probability of accepting to participate in COVID-19 vaccine trial and subsequently uptake the vaccine (i.e. being hesitant or not). In addition, the correlates of community members’ readiness to participate in a COVID-19 vaccine trial (Yes = 0 or No = 1) and uptake the COVID-19 vaccine (Yes = 0 or No = 1) were determined. The use of binary probit regression is justified because of the dichotomous nature of the dependent variables [19]. However, the binary probit regression is a nonlinear model, and its coefficients cannot tell us the magnitude of the effects of changes in the explanatory variables on an outcome variable [20]. Consequently, AMEs of the explanatory variables were used to make their interpretations intuitively meaningful. Moreover, AMEs instead of Marginal Effects at the Means (MEMs), were used because AMEs are deemed to be superior [21].

## Results

### Background information of respondents

Table 1 presents the background characteristics of respondents. A total of 2107 adult respondents aged 18 years and above attempted to answer the questionnaire. Out of this number, 1556 correctly completed and submitted the questionnaire, representing 74% response rate. Notwithstanding, some variables had missing responses. In terms of sex of respondents, 51% were males. The mean age was approximately 33 ± 10 years. Majority of respondents (68%) had at least tertiary education. Health workers (24%) and teachers (20%) dominated in the study participants, while artisans were the least category of workers. A higher proportion indicated they resided in Greater Accra (27%) and Volta regions (24%) at the time of responding to the questionnaire. Approximately 54% of the respondents reported they were not married, while 92% of them were Christians (see Table 1).

**Table 1:** Descriptive statistics on the socio-demographic characteristics of respondents

Characteristics	Freq. (f)	Percent (%)
Sex		
Male	777	50.65
Female	757	49.35
Total	1534	100.00
Age range		
18–23	197	13.63
24–29	374	25.88
30–35	454	31.42
36–41	232	16.06
42–47	85	5.88
48 +	103	7.13
Total	1445	100.00
Actual age (Mean, Std. Dev.)	32.77 (9.72)	
Education		
No formal education	204	13.65
Primary	55	3.68
Middle/JHS/JSS	50	3.34
Secondary/SHS/SSSS	171	11.44
Tertiary	1015	67.89
Total	1495	100.00
Occupation		
Artisan	72	4.93
Farmer	110	7.53
Teacher	295	20.19
Health worker	356	24.37
Trader	243	16.63
Other	385	26.35
Total	1461	100.00
Region		
Ashanti	93	6.28
Brong Ahafo	27	1.82
Bono East	47	3.17
Central	137	9.25
Eastern	147	9.93
Greater Accra	399	26.94
Northern	55	3.71
Oti	5	0.34
Upper East	31	2.09
Upper West	36	2.43
Volta	350	23.63
Western	132	8.91
Western North	22	1.49
Total	1481	100.00
Marital status		
Divorced	32	2.23
Living together	57	3.97
Married	663	46.20
Never married	642	44.74
Separated	21	1.46
Widowed	20	1.39
Total	1435	100.00
Religion		
Christian	1406	92.26
Moslem	98	6.43
Traditionalist/other	20	1.31
Total	1524	100.00
COVID-19 is caused by a virus		
No	47	3.42
Yes	1326	96.58
Total	1373	100.00
Participate in COVID-19 vaccine trial		
No	657	60.22
Yes	434	39.78
Total	1091	100.00
Advise someone to participate in a COVID-19 vaccine		
No	579	53.96
Yes	494	46.04
Total	1073	100.00
Accept to be immunized with a COVID-19 vaccine		
No	380	35.28
Yes	697	64.72
Total	1077	100.00
Advice someone to take COVID-19 vaccine		
No	338	31.24
Yes	744	68.76
Total	1082	100.00
Willing to pay for COVID-19 vaccine		
No	628	58.36
Yes	448	41.64
Total	1076	100.00
Amount willing to pay (Mean, Std. Dev.)	38.82 (80.53)	

Source: Field Data (2020); *COVID-19* Coronavirus Disease 2019, *JHS* Junior High School, *SHS* Senior High School, *JSS* (Junior Secondary School, *SSS* Senior Secondary School

### Perceptions on COVID-19 and the vaccine

About 97% of the respondents agreed that COVID-19 is caused by a virus. However, when they were asked whether they will be willing to participate in COVID-19 vaccine trail, 60% said they will not participate in a COVID-19 vaccine trial, and 54% of the respondents said they will advise someone to participate in a COVID-19 vaccine trial. Approximately 65% of respondents said they will accept to be immunized with a COVID-19 vaccine if it was introduced; 68% said they will advise someone to take the vaccine. With regard to payment for COVID-19 vaccine, 58% of respondents were not willing to pay for a COVID-19 vaccine when available. However, respondents who were willing to pay for COVID-19 vaccine said they were willing to pay approximately US$7.0 for full vaccination (see Table 1).

**Fig. 1:**
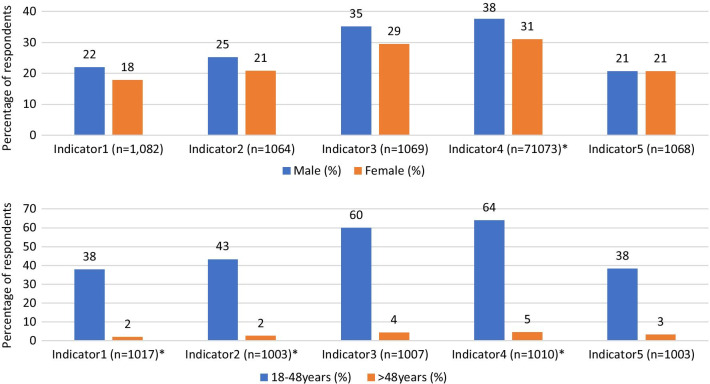
Perceptions on COVID-19 vaccination: disaggregated by gender and age. Source: (Field Data, 2020); Legend: Indicator1 (Participate in COVID-19 vaccine trial); Indicator2 (Advise someone to participate in COVID-19 vaccine trial); Indicator3 (Accept/take vaccination for COVID-19); Indicator4 (Advise someone to accept/take COVID-19 vaccination); Indicator5 (Willingness to pay for COVID-19 vaccination); *Chi-square test statistically significant at 95% confidence level

In terms of respondent’s sex differentials, it was observed that more males (38%) than females (31%) will advise someone to take the COVID-19 vaccine (*p* < 0.05). Younger respondents (aged 18–47 years) said they will participate in COVID-19 vaccine trial, advise someone to participate in COVID-19 vaccine trial as well as eventually take the vaccine than older respondents (> 48 years) (*p* < 0.05) (see Fig. 1).

**Fig. 2:**
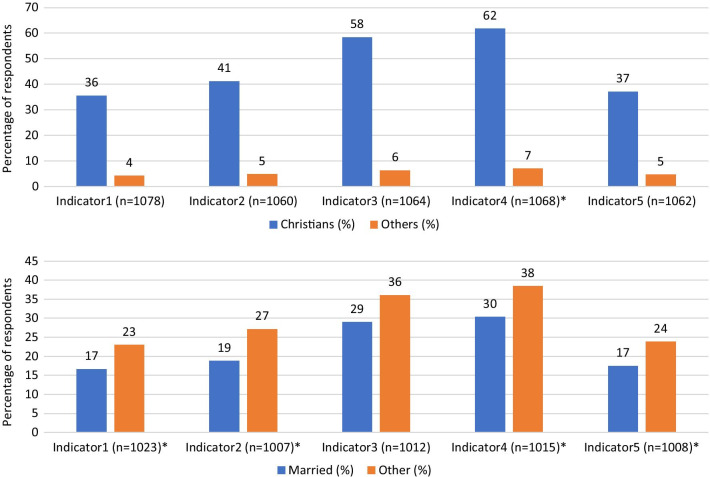
Perceptions on COVID-19 vaccination: disaggregated by religion and marital status. Source: Field Data (2020); Legend: Indicator1 (Participate in COVID-19 vaccine trial); Indicator2 (Advise someone to participate in COVID-19 vaccine trial); Indicator3 (Accept/take vaccination for COVID-19); Indicator4 (Advise someone to accept/take COVID-19 vaccination); Indicator5 (Willingness to pay for COVID-19 vaccination); *Chi-square test statistically significant at 95% confidence level

**Fig. 3:**
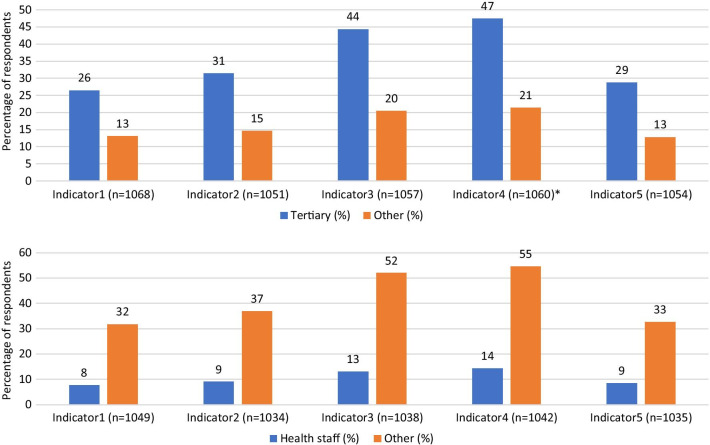
Perceptions on COVID-19 vaccination: disaggregated by education and occupation. Source: Field Data (2020); Legend: Indicator1 (Participate in COVID-19 vaccine trial); Indicator2 (Advise someone to participate in COVID-19 vaccine trial); Indicator3 (Accept/take vaccination for COVID-19); Indicator4 (Advise someone to accept/take COVID-19 vaccination); Indicator5 (Willingness to pay for COVID-19 vaccination); *Chi-square test statistically significant at 95% confidence level

It was also observed that more unmarried respondents said they will participate in a COVID-19 vaccine trial (23%), and advise others to do same (27%) than married individuals (*p* < 0.05). Furthermore, more Christians (62%) than other religious affiliations indicated they will advise someone to take the COVID-19 vaccine (*p* < 0.05) (see Fig. 2). In addition, respondents with at least tertiary education reported they will advise someone to accept the COVID-19 vaccine than persons with lower educational qualifications (*p* < 0.05) (see Fig. 3).

### Predictors of voluntary participation in COVID-19 vaccine trial

Binary probit regression (with AMEs) was conducted to determine the correlates of respondents’ willingness to participate in a COVID-19 vaccine trial and recommend same to others. It was observed that females relative to males, were on average 6% times less likely to voluntarily participate in COVID-19 vaccine trial (AME = − 0.06, SE = 0.03, *p* < 0.1). Relative to persons aged 18–23 years, the percentage chance of participating in COVID-19 vaccine trial was on average less among 48 + year-olds (AME = − 0.35, SE = 0.07, *p* < 0.01), followed by persons aged 36–41 years (AME = − 0.23, SE = 0.07, *p* < 0.01), 42–47 years (AME = − 0.15, SE = 0.09, *p* < 0.1), 30–35 years (AME = − 0.15, SE = 0.06, *p* < 0.05) and 24–29 years (AME = − 0.13, SE = 0.06, *p* < 0.05). Similar age differentials were found with regard to recommending COVID-19 vaccine trial to someone.

Regional differentials show that, on average, relative to Ashanti region, residents of Oti region had a higher percentage chance of participating in a COVID-19 vaccine trial (AME = 0.51, SE = 0.23, *p* < 0.05), followed by Upper East (AME = 0.35, SE = 0.15, *p* < 0.05), Upper West (AME = 0.23, SE = 0.11, *p* < 0.05), Central (AME = 0.22, SE = 0.08, *p* < 0.01), Northern (AME = 0.20, SE = 0.10, *p* < 0.1), Eastern (AME = 0.18, SE = 0.07, *p* < 0.05) and Volta regions (AME = 0.12, SE = 0.06, *p* < 0.1). Differences in the other regions were not statistically significant. However, similar regional differentials were observed concerning recommending COVID-19 vaccine trial to someone. Respondents willing to pay for COVID-19 vaccine were on average, 25% times (AME = 0.25, SE = 0.03, *p* < 0.01) and 27% times (AME = 0.27, SE = 0.03, *p* < 0.01) more likely to participate in and recommend COVID-19 vaccine trial, respectively, relative to those unwilling to pay (see Table 2).

**Table 2:** Probit regression estimates of determinants of willingness to participate in COVID-19 vaccine trial

	Dependent variable: participation in COVID-19 vaccine trial	
	Self^+^	Someone^++^
Independent variables	AME(SE)	AME(SE)
Sex (Ref: male)		
Female	− 0.06^*^ (0.03)	− 0.05 (0.03)
Age (Ref: 18–23)		
24–29	− 0.13^**^ (0.06)	− 0.16^***^ (0.06)
30–35	− 0.15^**^ (0.06)	− 0.16^***^ (0.06)
36–41	− 0.23^***^ (0.07)	− 0.18^***^ (0.07)
42–47	− 0.15^*^ (0.09)	− 0.08 (0.09)
48 +	− 0.35^***^ (0.07)	− 0.28^***^ (0.08)
Education (Ref: none)		
Primary	− 0.08 (0.08)	− 0.05 (0.08)
Middle/JHS/JSS	0.14 (0.09)	0.07 (0.09)
Secondary/SHS/SSSS	0.05 (0.07)	0.02 (0.07)
Tertiary	− 0.01 (0.05)	0.02 (0.05)
Region (Ref: Ashanti)		
Brong Ahafo	0.12 (0.11)	0.15 (0.12)
Bono East	0.13 (0.12)	0.24^**^ (0.12)
Central	0.22^***^ (0.08)	0.27^***^ (0.08)
Eastern	0.18^**^ (0.07)	0.26^***^ (0.07)
Greater Accra	0.09 (0.06)	0.12^**^ (0.06)
Northern	0.20^*^ (0.10)	0.25^**^ (0.10)
Oti	0.51^**^ (0.23)	0.45^*^ (0.25)
Upper East	0.35^**^ (0.15)	0.33^**^ (0.15)
Upper West	0.23^**^ (0.11)	0.26^**^ (0.11)
Volta	0.12^*^ (0.06)	0.20^***^ (0.06)
Western	0.11 (0.08)	0.17^**^ (0.08)
Western North	0.21 (0.13)	0.21 (0.13)
Marital status (Ref: divorced)		
Living together	− 0.09 (0.13)	0.05 (0.13)
Married	0.00 (0.11)	0.07 (0.11)
Never married	− 0.03 (0.12)	0.11 (0.12)
Separated	0.05 (0.18)	0.08 (0.18)
Widowed	0.20 (0.17)	0.18 (0.17)
Religion (Ref: Christian)		
Moslem	− 0.03 (0.06)	0.00 (0.06)
Traditionalist/other	− 0.00 (0.13)	− 0.08 (0.13)
Willingness to pay (Ref: no)		
Yes	0.25^***^ (0.03)	0.27^***^ (0.03)
COVID-19 is caused by a virus (Ref: no)		
Yes	− 0.09 (0.09)	− 0.07 (0.09)
Observations chi2	911	903

Source: Field Data (2020); *COVID-19* Coronavirus Disease 2019, *JHS* (unior High School, *SHS* Senior High School, *JSS* Junior Secondary School, *SSS* Senior Secondary School, *AME* Average Marginal Effect, Average marginal effects; Standard errors in parentheses; *p < 0.1, **p < 0.05, ***p < 0.01; ^+^Self (the respondents himself or herself); ^++^ (someone else including friends, relatives and co-workers etc.)

### Determinants of COVID-19 vaccine uptake

It was found that, on average, relative to respondents aged 18–23 years, respondents aged 48 years and above had a higher percentage chance of not accepting the COVID-19 vaccine (AME = − 0.21, SE = 0.08, *p* < 0.01), followed by 36–41 years (AME = − 0.18, SE = 0.06, *p* < 0.01); 24–29 years (AME = − 0.17, SE = 0.05, *p* < 0.01); 42–47 years (AME = − 0.15, SE = 0.08, *p* < 0.05) and 30–35 years (AME = − 0.13, SE = 0.05, *p* < 0.05).

It was also observed that relative to persons without formal education, respondents with at most Middle or Junior High School education were less likely to accept the COVID-19 vaccine (AME = − 0.18, SE = 0.09, *p* < 0.05), followed by respondents who attained primary school level (AME = − 0.16, SE = 0.08, *p* < 0.05) and secondary school level (AME = − 0.13, SE = 0.06, *p* < 0.05).

In terms of the regional differentials, relative to Ashanti region, willingness to accept the COVID-19 vaccine was significantly highest among respondents from Upper West region (AME = 0.31, SE = 0.09, *p* < 0.01) followed by Northern (AME = 0.26, SE = 0.09, *p* < 0.01), Western North (AME = 0.24, SE = 0.13, *p* < 0.1), Western (AME = 0.23, SE = 0.08, *p* < 0.01), Eastern (AME = 0.21, SE = 0.07, *p* < 0.01), Central (AME = 0.20, SE = 0.07, *p* < 0.01) and Volta regions (AME = 0.11, SE = 0.06, *p* < 0.1). Differences in the other regions were not statistically significant. Concerning recommending to someone to uptake the vaccine, similar regional differences were observed.

On average, Moslems were 12% times less likely to accept COVID-19 vaccine relative to Christians (AME = − 0.12, SE = 0.06, *p* < 0.1). Respondents who were willing to pay for COVID-19 vaccine were 34% and 35% times more likely to accept COVID-19 vaccine (AME = 34, SE = 0.03, *p* < 0.01) and recommend it to someone (AME = 35, SE = 0.03, *p* < 0.01), respectively, than those not willing to pay for the vaccine.

However, there was no statistically significant association between knowledge of COVID-19 and willingness to accept COVID-19 vaccine. Likewise, marital status did not have a statistically significant association with willingness to take COVID-19 vaccine (see Table 3).

**Table 3:** Probit regression estimates of determinants of willingness to accept or uptake COVID-19 vaccine

		Dependent variable: accept COVID-19 vaccine
	Self^+^	Someone^++^
Independent variables	AME(SE)	AME(SE)
Sex (Ref: male)		
Female	− 0.05 (0.03)	− 0.06^**^ (0.03)
Age (Ref: 18–23)		
24–29	− 0.17^***^ (0.05)	− 0.09^*^ (0.05)
30–35	− 0.13^**^ (0.05)	− 0.04 (0.05)
36–41	− 0.18^***^ (0.06)	− 0.07 (0.06)
42–47	− 0.15^**^ (0.08)	0.00 (0.07)
48 +	− 0.21^***^ (0.08)	− 0.12 (0.08)
Education (Ref: none)		
Primary	− 0.16^**^ (0.08)	− 0.11 (0.07)
Middle/JHS/JSS	− 0.18^**^ (0.09)	− 0.18^**^ (0.08)
Secondary/SHS/SSSS	− 0.13^**^ (0.06)	− 0.14^**^ (0.06)
Tertiary	− 0.07 (0.04)	− 0.07 (0.04)
Region (Ref: Ashanti)		
Brong Ahafo	0.16 (0.11)	0.23^**^ (0.11)
Bono East	0.15 (0.12)	0.16 (0.11)
Central	0.20^***^ (0.07)	0.27^***^ (0.07)
Eastern	0.21^***^ (0.07)	0.26^***^ (0.07)
Greater Accra	0.10 (0.06)	0.16^**^ (0.06)
Northern	0.26^***^ (0.09)	0.24^**^ (0.10)
Oti	0.20 (0.25)	0.20 (0.25)
Upper East	0.16 (0.14)	0.19 (0.14)
Upper West	0.31^***^ (0.09)	0.31^***^ (0.09)
Volta	0.11^*^ (0.06)	0.20^***^ (0.06)
Western	0.23^***^ (0.08)	0.23^***^ (0.08)
Western North	0.24^*^ (0.13)	0.24^**^ (0.12)
Marital status (Ref: divorced)		
Living together	0.02 (0.13)	0.16 (0.13)
Married	0.03 (0.11)	0.12 (0.11)
Never married	0.03 (0.11)	0.16 (0.12)
Separated	0.17 (0.15)	0.30^**^ (0.14)
Widowed	− 0.15 (0.16)	− 0.01 (0.17)
Religion (Ref: Christian)		
Moslem	− 0.12^*^ (0.06)	− 0.06 (0.06)
Traditionalist/other	0.08 (0.12)	0.19^*^ (0.10)
Willingness to pay (Ref: no)		
Yes	0.34^***^ (0.03)	0.35^***^ (0.03)
COVID-19 is caused by a virus (Ref: no)		
Yes	0.13 (0.08)	0.07 (0.08)
Observations	905	906

Source: Field Data (2020); *COVID-19* Coronavirus Disease 2019, *JHS* Junior High School, *SHS* Senior High School, *JSS* Junior Secondary School, *SSS* Senior Secondary School, Average Marginal Effects; Standard errors in parentheses; *p < 0.1, **p < 0.05, ***p < 0.01; ^+^Self (the respondents himself or herself); ^++^(someone else including friends, relatives and co-workers etc.)

Predominant reasons cited for hesitancy in participating in a COVID-19 vaccine trial and eventually taking the vaccine when given the opportunity included fear, safety concerns, lack of trust, uncertainty, political connotations, spiritual and religious beliefs (see Additional file 1 for details).

## Discussion

According to the WHO, 59.3 million doses of the COVID-19 vaccines had already been distributed and 39 million administered in the United States alone as of February 8, 2021 [22]. Globally, over 400 million doses are said to have been administered as of March, 2021 [23]. Even though there have been reports of some adverse events from the COVID-19 vaccines, available evidence suggests the benefits of the vaccine in terms of reducing mortalities from COVID-19, far outweigh these adverse events1 [24]. In Ghana, an estimated 400,000 persons have already received the first dose of the COVID-19 vaccine by the second week of March, 2021.

Vaccines are an important intervention for public health emergencies but acceptability and willingness to uptake same have always been challenged by misconceptions and conspiracy theories resulting in vaccine hesitancy. Concerns and fears on the safety of the COVID-19 vaccines are especially widespread in Ghana and other countries because of the record time discovery of the vaccine, the lack of trust in governments’ response, secrecy and the misinformation surrounding the pandemic [25]

It was found that many of the participants were risk-averse. For instance, we found that 60% of respondents said they will not volunteer to participate in a COVID-19 vaccine trial similar to a study by Goldman et al. [26] among caregivers in six countries. A systematic review by Sallam [27] showed vaccine acceptance rate in 33 different countries ranged from a high of 97% in Ecuador to a low of 58.9% in France, thus placing Ghana in an above-average acceptance rate. It was also found in this study that 69% of the people said they will recommend the COVID-19 vaccine to a friend or relative, suggesting a high level of confidence and trust in the vaccine contrary to the media reportage in Ghana that confidence and trust in the vaccine is low. Media reportage has been found to play a critical role in vaccine hesitancy in many countries [28] and must, therefore, be targeted in the vaccine campaign strategies.

Willingness to pay for COVID-19 vaccine was, however, found to be low as 58% of respondents said they will be willing to pay at most $7.0 for a COVID-19 vaccine. Even though the current vaccine is free for persons in Ghana, this revelation is important, since it demonstrates the level of commitment for future cost-sharing for the vaccine as the Government of Ghana (GoG) gets weaned off from assistantship with regards to vaccines [29]. Moreover, subsequent consignments of the COVID-19 vaccine being procured for deployment are at a cost which has started raising public debate on whether government can continue absorb these costs. Since, this study did not exhaustively explore willingness to pay for the COVID-19 vaccine, it is recommended that future research endeavours investigate this important grey area to adduce empirical evidence that will inform national policy dialogue on financing of vaccines in Ghana and Africa at large.

Another observation from this study was the significant age and sex differences in willingness to participate in COVID-19 vaccine trials and to take the vaccine. More males expressed readiness to participate in COVID-19 vaccine trial and to take the vaccine than their female counterparts. In addition, younger persons indicated the willingness to participate in COVID-19 vaccine trials and take the vaccines compared to the older counterparts. Likewise, religious affiliation and marital status significantly correlate with either volunteering for COVID-19 vaccine trial, willingness to take the vaccine or otherwise.

These observations suggest the need to adequately engage these segments of society in the rollout plan for the vaccine. In addition, the results corroborate findings by similar studies on vaccine hesitancy [26, 27, 30] which found that sex, age and religion are important predictors of willingness to uptake the COVID-19 vaccine. A relatively lower acceptance rate by elderly people was rather counter-intuitive, since this category of people are more at risk of complications from COVID-19 infection. Targeted public health education is, therefore, necessary to reach out to this category of persons in society. Perhaps the current public health communication channels are not inclusive enough for the elderly. Targeted vaccine campaigns in Moslem populated communities will also help address the relatively higher COVID-19 vaccine hesitancy among adult Moslem populations as observed in this study.

Counter-intuitively, it was found that hesitancy for COVID-19 vaccine trial and the vaccine itself was high in regions which were epicentres of COVID-19 pandemic and more urbanized. Perhaps the relatively higher exposure of persons living in the bigger cities to diverse social media-information (some of which could be misleading and anti-vaccine) could have sensitised residents in these high-risk areas against the vaccine. For instance, Puri et al. [28] alluded to the negative effect of social media on uptake of vaccines, including the COVID-19 vaccine. To help address this challenge, the Ministry of Health (MoH) and Ghana Health Service (GHS) have to re-strategise health campaigns in cosmopolitan regions. Vaccine campaign messages should equally target persons with relatively lower formal education as this category of persons within the population were less likely to voluntarily participate in a COVID-19 vaccine trial, uptake the vaccine and recommend it to someone. Association between educational qualification and willingness to uptake COVID-19 vaccine is varied as per the available empirical literature observed outside [8–11, 13–15, 26, 28, 30] and within Africa [16, 17]. As study by Kanyike et al. [31] found that barely 37% of 600 medical students interviewed in Uganda will accept COVID-19 vaccine. This COVID-19 vaccine acceptance rate is one of the lowest in Africa, especially among persons with privileged medical knowledge than the general population.

More importantly, fear, lack of trust, concerns on vaccine safety must be addressed through effective public health campaigns leveraging existing social media platforms that are effective and trusted. Re-tooling the Information Services Department and the National Commission for Civic Education to communicate these health campaigns in local languages and dialects at the community level will also help address the existing information gaps on the COVID-19 vaccine to reduce hesitancy and promote acceptance of the vaccine. These interventions, in addition to ongoing efforts will help realise Ghana’s target of vaccinating at least 20 million people.

## Limitations

First, the survey was conducted via an online platform mostly deployed via social media platforms. This approach could have created possible selection bias, where people who are Information Technology (IT) savvy participated more in the study. This approach could have inadvertently excluded persons without formal education or non-ownership of smartphones to access the questionnaire. In anticipation of this limitation, the researchers trained community health nurses (mostly working in primary healthcare facilities at the community level across the country) to administer the tool to persons who were illiterates or did not have access to smartphones and internet service. Another limitation is that the study was conducted during the trial periods of the COVID-19 vaccines when their efficacy as well as side effects were unknown. Perhaps if respondents are to be interviewed now, their responses may be different since there is now more information on the vaccines in terms of their efficacy and side effects.

## Conclusion

Acceptance rate for COVID-19 vaccine in Ghana is relatively high (65%) compared to countries, such as DRC (27.7%) and France (58.9%). Willingness to pay for the vaccine was, however, low with barely 58% of respondents willing to pay an average of $7.0 for a vaccine. Significant predictors of the COVID-19 vaccine uptake were gender, age, region of residence, education level and willingness to pay for the cost of the vaccine. These significant dynamics must, therefore, be taken into consideration as part of the vaccine rollout strategies to achieve wider coverage.

## Recommendations

While acknowledging the limitations associated with this study, the following policy recommendations are proposed. First, health campaigns on COVID-19 vaccine uptake must address concerns of fear, mistrust and safety concerns to promote acceptance of the COVID-19 vaccine. Second, there is the need for targeted vaccine uptake messages taking into consideration the role of gender, age and educational dynamics to promote acceptance of the vaccine. Moreover, religious institutions and their leadership should be actively engaged in public health education campaigns to demystify the vaccine and engender trust and confidence. Finally, the regional differentials in willingness to uptake the vaccine suggest the need for region-specific health education approaches to address their peculiar concerns.

## Supplementary Information

**Additional file 1. Table S1.** Verbatim reasons cited for unwillingness to participate in a COVID-19 vaccine Trial.

## Data Availability

There are no restrictions to data and materials used in this manuscript.

## References

[1] WHO Coronavirus Disease (COVID-19) Dashboard. https://covid19.who.int/?clid=cjwkcajwxev3brbbeiwaib_pwbixaebnx4l6jsavlqdk4b77sjpknrmtakemaqoywnrpdcrvvjjdxrocrgeqavd_bwe . Accessed 13 Jul 2021.

[2] Patterson KD (1983). The influenza epidemic of 1918–19 in the Gold Coast. J Afr Hist.

[3] Ghana Health Service (GHS). Official website, 2021. https://www.ghanahealthservice.org/covid19/. Accessed 13 Jul 2021.

[4] WHO Official Website. Coronavirus 2019 vaccines. 2021. https://www.who.int/emergencies/diseases/novel-coronavirus-2019/covid-19-vaccines. Accessed 13 Jul 2021.

[5] WHO Official Website. COVID-19 vaccine doses shipped by the COVAX Facility head to Ghana, marking beginning of global rollout. 2021. https://www.who.int/news/item/24-02-2021-covid-19-vaccine-doses-shipped-by-the-covax-facility-head-to-ghana-marking-beginning-of-global-rollout. Accessed 13 Jul 2021.

[6] DW. Official Website. Top stories: Coronavirus: Ghana's president gets first COVID jab on 1st March, 2021. https://www.dw.com/en/coronavirus-ghanas-president-gets-first-covid-jab/a-56734567. Accessed 14 Mar 2021.

[7] GhanaWeb Official Website. Editorial News of Wednesday, 10 March 2021. https://www.ghanaweb.com/GhanaHomePage/NewsArchive/The-Herald-Ghana-Kojo-Oppong-Nkrumah-needs-to-grow-up-1200805. Accessed 14 Mar 2021.

[8] Malik AA, McFadden SM, Elharake J, Omer SB (2020). Determinants of COVID-19 vaccine acceptance in the US. EClinicalMedicine.

[9] Reiter PL, Pennell ML, Katz ML (2020). Acceptability of a COVID-19 vaccine among adults in the United States: How many people would get vaccinated?. Vaccine.

[10] Kuter BJ, Browne S, Momplaisir FM, Feemster KA, Shen AK, Green-McKenzie J, Faig W, Offit PA (2021). Perspectives on the Receipt of a COVID-19 vaccine: a survey of employees in two large hospitals in Philadelphia. Vaccine.

[11] Fisk RJ (2021). Barriers to vaccination for COVID-19 control—experience from the United States. Glob Health J.

[12] Nguyen KH, Srivastav A, Razzaghi H, Williams W, Lindley MC, Jorgensen C, Abad N, Singleton J (2021). COVID-19 Vaccination Intent, Perceptions, and Reasons for Not Vaccinating Among Groups Prioritized for Early Vaccination—United States, September and December 2020. Morb Mortal Wkly Rep.

[13] Wang J, Jing R, Lai X, Zhang H, Lyu Y, Knoll MD, Fang H (2020). Acceptance of COVID-19 vaccination during the COVID-19 pandemic in China. Vaccines.

[14] Chen M, Li Y, Chen J, Wen Z, Feng F, Zou H, Fu C, Chen L, Shu Y, Sun C (2021). An online survey of the attitude and willingness of Chinese adults to receive COVID-19 vaccination. Hum Vaccin Immunother.

[15] Alqudeimat Y, Alenezi D, AlHajri B, Alfouzan H, Almokhaizeem Z, Altamimi S, Almansouri W, Alzalzalah S, Ziyab AH (2021). Acceptance of a COVID-19 vaccine and its related determinants among the general adult population in Kuwait. Med Princ Pract.

[16] Ditekemena JD, Nkamba DM, Mavoko AM, Hypolite M, Siewe Fodjo JN, Luhata C, Obimpeh M, Van Hees S, Nachega JB, Colebunders R (2021). COVID-19 vaccine acceptance in the democratic republic of congo: a cross-sectional survey. Vaccines.

[17] Afolabi AA, Ilesanmi OS (2021). Dealing with vaccine hesitancy in Africa: the prospective COVID-19 vaccine context. Pan Afr Med J.

[18] Krejcie RV, Morgan DW (1970). Determining sample size for research activities. Educ Psychol Measur.

[19] Cameron AC, Trivedi PK (2005). Microeconometrics: Methods and Applications.

[20] Cameron AC, Trivedi PK (2010). Microeconometrics Using Stata.

[21] Williams R (2012). Using the margins command to estimate and interpret adjusted predictions and marginal effects. Stand Genomic Sci.

[22] Reuters News: 59.3 million doses of COVID-19 vaccines distributed, 39 million administered: U.S. CDC, February 7, 2021. https://www.reuters.com/article/us-health-coronavirus-usa-cdc-idUSKBN2A701B. Accessed 24 Mar 2021.

[23] World Health Organization (WHO). Official Website: WHO Coronavirus (COVID-19) Dashboard. https://covid19.who.int/. Accessed 24 Mar 2021.

[24] Remmel A (2021). COVID vaccines and safety: what the research says. Nature.

[25] Bhopal S, Nielsen M (2021). Vaccine hesitancy in low- and middle-income countries: potential implications for the COVID-19 response. Arch Dis Child.

[26] Goldman RD, Marneni SR, Seiler M, Brown JC, Klein EJ, Cotanda CP, Gelernter R, Yan TD, Hoeffe J, Davis AL, Griffiths MA (2020). Caregivers' willingness to accept expedited vaccine research during the COVID-19 pandemic: a cross-sectional survey. Clin Ther.

[27] Sallam M (2021). COVID-19 vaccine hesitancy worldwide: a concise systematic review of vaccine acceptance rates. Vaccines.

[28] Puri N, Coomes EA, Haghbayan H, Gunaratne K (2020). Social media and vaccine hesitancy: new updates for the era of COVID-19 and globalized infectious diseases. Hum Vaccin Immunother.

[29] Deliotte. 2021 Government of Ghana Budget Statement and Economic Policy: Summary of budget statement & Deloitte views. 2021. https://www2.deloitte.com/content/dam/Deloitte/gh/Documents/tax/DELOITTE%20BUDGET%20REVIEW%202021.pdf. Accessed 18 May 2021.

[30] Rutten LJF, Zhu X, Leppin AL, Ridgeway JL, Swift MD, Griffin JM, St Sauver JL, Virk A, Jacobson RM. Evidence-based strategies for clinical organizations to address COVID-19 vaccine hesitancy. In: Mayo Clinic Proceedings. Elsevier; 2020. 10.1016/j.mayocp.2020.12.024 PMC7772995 33673921

[31] Kanyike AM, Olum R, Kajjimu J, Ojilong D, Akech GM, Nassozi DR, Agira D, Wamala NK, Asiimwe A, Matovu D, Nakimuli AB, Lyavala M, Kulwenza P, Kiwumulo J, Bongomin F (2021). Acceptance of the coronavirus disease-2019 vaccine among medical students in Uganda. Trop Med Health..

