# Supplementary material for: COVID-19 vaccine hesitancy among the adult population in Ghana: evidence from a pre-vaccination rollout survey
Source: Trop Med Health. 2021 Dec 16;49:96. doi: 10.1186/s41182-021-00357-5 (PMC8674411; doi:10.1186/s41182-021-00357-5)
Supplement: Supplementary file 1 — Additional file 1. Table S1. Verbatim reasons cited for unwillingness to participate in a COVID-19 vaccine Trial. [file 41182_2021_357_MOESM1_ESM.rtf]

Table 1: Verbatim reasons cited for unwillingness to participate in a COVID-19 vaccine Trial 
Specify reasons you would or wouldn't participate in COVID-19 vaccine trial	Freq.	Percent	Cum.	
Afraid	9	1.61	1.61	
Afraid of vaccine trial side effects	1	0.18	1.79	
Africa is not a shit hole	1	0.18	1.97	
Am afraid	3	0.54	2.51	
Am afraid	3	0.54	3.05	
Am afraid of having a reaction	1	0.18	3.23	
Am afraid to it will give me health problems	1	0.18	3.41	
Am nervous	1	0.18	3.58	
Am not 100% sure it has been tested well.	1	0.18	3.76	
Am not a health practitioner, perhaps I have no knowledge concerning drugs and medicine	1	0.18	3.94	
Am not a medical expert	1	0.18	4.12	
Am not a test subject.	1	0.18	4.30	
Am not infected	1	0.18	4.48	
Am not interested	1	0.18	4.66	
Am not ok with vaccine	1	0.18	4.84	
Am not sick	1	0.18	5.02	
Am not sick of the virus	1	0.18	5.20	
Am not sure of the outcome	1	0.18	5.38	
Am not sure of the vaccine	1	0.18	5.56	
Am old	1	0.18	5.73	
Am scared	4	0.72	6.45	
Anxious	1	0.18	6.63	
Any adverse effects on me could kill me since there would not be enough attention to treat me. I am an ordinary Ghanaian and attention is not paid to us in times of troubles	1	0.18	6.81	
Because I do not show any symptoms	1	0.18	6.99	
Because I don't believe in it	1	0.18	7.17	
Because I don't believe in that medicine	1	0.18	7.35	
Because I don't believe those vaccines	1	0.18	7.53	
Because I don't have Corona	1	0.18	7.71	
Because I don't have any idea about the vaccine	1	0.18	7.89	
Because I don't have the disease	2	0.36	8.24	
Because I don't know the side effect	1	0.18	8.42	
Because I don't know the side effects	1	0.18	8.60	
Because I don't trust the vaccine	1	0.18	8.78	
Because I don't want to	1	0.18	8.96	
Because I have never had any of the covid symptoms	1	0.18	9.14	
Because I have no COVID-19 so why should I be used for trial?	1	0.18	9.32	
Because I just won't do it	1	0.18	9.50	
Because I will not allow that	1	0.18	9.68	
Because I'm not a patient of the virus	1	0.18	9.86	
Because am I afear	1	0.18	10.04	
Because am not a victim of COVID- 19	1	0.18	10.22	
Because am not sick	1	0.18	10.39	
Because anything which is on trial basis it's not setting to be used on human. I will only participate after it has been confirmed to be used worldwide.	1	0.18	10.57	
Because I'm scared. I did rather stay home	1	0.18	10.75	
Because if it should fail and have risky implications on my life, I risk leaving my beneficiaries uncared for	1	0.18	10.93	
Because is not tested for its efficacy	1	0.18	11.11	
Because it is a gamble to begin with and it is probable that it may go wrong.	1	0.18	11.29	
Because it is too deadly	6	1.08	12.37	
Because it is trial	1	0.18	12.54	
Because it is not proved safe	1	0.18	12.72	
Because it's not safe	1	0.18	12.90	
Because l am scared	1	0.18	13.08	
Because of fear	2	0.36	13.44	
Because of my age	3	0.54	13.98	
Because of side effects and stigma	1	0.18	14.16	
Because the case fatality is high so i will not risk my life	1	0.18	14.34	
Because there is no sureness on the vaccine	1	0.18	14.52	
Because there is no vaccine	1	0.18	14.70	
Because they have to try it on their people first	1	0.18	14.87	
Because those tasted, some have died	1	0.18	15.05	
Because we are not sure of it infect in human systems	1	0.18	15.23	
Because we heard it is not safe	1	0.18	15.41	
Because you are not sure if it's safe	1	0.18	15.59	
Best known to me	1	0.18	15.77	
Because the authenticity not guaranteed	1	0.18	15.95	
Busy at work	1	0.18	16.13	
Can kill	1	0.18	16.31	
Cannot trust the source	1	0.18	16.49	
Can risk myself	1	0.18	16.67	
Can trust the white	1	0.18	16.85	
Can't take such risk	1	0.18	17.03	
Cannot use me for trial	1	0.18	17.20	
Can't tell	1	0.18	17.38	
Concerned about safety	10	1.79	19.18	
Could cause illness	1	0.18	19.35	
Dangerous	1	0.18	19.53	
Do not have time	1	0.18	19.71	
Do not trust the vaccine	2	0.36	20.07	
Do not trust the vaccine trial	8	1.43	21.51	
Do you want to kill me?	1	0.18	21.68	
Don't know effect of the new vaccine	1	0.18	21.86	
Don't know the side effects of the vaccine	1	0.18	22.04	
Don't know yet	1	0.18	22.22	
Don't like to take risk	1	0.18	22.40	
Don't no effect of the vaccine	1	0.18	22.58	
Don't trust in the vaccine	1	0.18	22.76	
Don't trust the system	1	0.18	22.94	
Don't trust the vaccines unless proven otherwise	1	0.18	23.12	
Don't want to be used as a lab rat	1	0.18	23.30	
Don't want to be used for an experiment	1	0.18	23.48	
Don't know how effective it is	1	0.18	23.66	
Don't know whether it good for me	1	0.18	23.84	
Don't want anything to do with Corona virus	1	0.18	24.01	
Don't want to take risk	2	0.36	24.37	
Due to try and error. Yet to be determined	1	0.18	24.55	
Fear reason	1	0.18	24.73	
Fear	27	4.84	29.57	
Fear if the unknown	1	0.18	29.75	
Fear of side effects	1	0.18	29.93	
Fear of some complications	1	0.18	30.11	
Fear of stigmatization	1	0.18	30.29	
Fear of the unknown as people have died of testing vaccines	1	0.18	30.47	
Fear of unknown side effects	1	0.18	30.65	
Fear the western world	1	0.18	30.82	
For credibility reasons	1	0.18	31.00	
Had a relative who had a bad experience with Hep B vaccine	1	0.18	31.18	
Have allergies	1	0.18	31.36	
Have an underlying sickness	1	0.18	31.54	
Health reasons	1	0.18	31.72	
How sure I'm I, if that thing is not deadly	1	0.18	31.90	
How will you know it's perfect?	1	0.18	32.08	
I am a trial body	1	0.18	32.26	
I am afraid	1	0.18	32.44	
I am afraid	2	0.36	32.80	
I am afraid of injection	1	0.18	32.97	
I am not Tweneboah Koduah	1	0.18	33.15	
I am not a thing to be experimented on.	1	0.18	33.33	
I am not a victim	1	0.18	33.51	
I am not infected	2	0.36	33.87	
I am not infected with the virus	1	0.18	34.05	
I am not interested	2	0.36	34.41	
I am not ready mentally to test	1	0.18	34.59	
I am not sick	2	0.36	34.95	
I believe trials should be done on animals who whose immune system are similar with ours	1	0.18	35.13	
I can die	1	0.18	35.30	
I can only live once; I don't want to gamble on a drug I be no idea about its effect.	1	0.18	35.48	
I can't risk it	1	0.18	35.66	
I cannot trust it safety	1	0.18	35.84	
I can't risk it	1	0.18	36.02	
I can't sustain trials	1	0.18	36.20	
I don't trust the efficacy	1	0.18	36.38	
I don't want to be infected	1	0.18	36.56	
I do not know the effect on me	1	0.18	36.74	
I do not like trial things.	1	0.18	36.92	
I do not trust the white man, taking into account some deadly viruses they have introduced in Africa. I am not an experimental guinea pig.	1	0.18	37.10	
I don't believe in it	1	0.18	37.28	
I don't believe in the vaccine which will be used	1	0.18	37.46	
I don't feel it's safe yet	1	0.18	37.63	
I don't feel okay	1	0.18	37.81	
I don't have that confidence	1	0.18	37.99	
I don't have the disease	1	0.18	38.17	
I don't in it	1	0.18	38.35	
I don't know how I will react to it.	1	0.18	38.53	
I don't know how my body would adjust	1	0.18	38.71	
I don't know the actual side effects.	1	0.18	38.89	
I don't know what the outcome will be	1	0.18	39.07	
I don't like it	1	0.18	39.25	
I don't really know much about it please	1	0.18	39.43	
I don't see it right	1	0.18	39.61	
I don't think is healthy	1	0.18	39.78	
I don't trust any covid19 vaccine	1	0.18	39.96	
I don't trust it	1	0.18	40.14	
I don't trust it especially the side effect	1	0.18	40.32	
I don't trust our leaders	1	0.18	40.50	
I don't trust the system	1	0.18	40.68	
I don't trust the system	1	0.18	40.86	
I don't trust the test kits and the process	1	0.18	41.04	
I don't trust the vaccine	2	0.36	41.40	
I don't trust the vaccine	3	0.54	41.94	
I don't trust the vaccines	1	0.18	42.11	
I don't trust the western world	1	0.18	42.29	
I don't trust the white anymore	1	0.18	42.47	
I don't trust the white man's medicine	1	0.18	42.65	
I don't trust the white man, I may die if the drug fails or development some serious condition.	1	0.18	42.83	
I don't trust where the vaccine is coming from.	1	0.18	43.01	
I don't want any risk	1	0.18	43.19	
I don't want it.	1	0.18	43.37	
I don't want to	1	0.18	43.55	
I don't want to be infected	1	0.18	43.73	
I don't want to be used for experiment	1	0.18	43.91	
I don't want to be used for trials	1	0.18	44.09	
I don't want to die	10	1.79	45.88	
I don't want to die ??	1	0.18	46.06	
I don't want to endanger my life	1	0.18	46.24	
I don't want to have any side effect of any vaccine which doesn't have any prove of cure of the virus	1	0.18	46.42	
I don't want to waste my time	1	0.18	46.59	
I don't why should be considered for vaccine trail	1	0.18	46.77	
I don't have symptoms of COVID-19	1	0.18	46.95	
I don't know where they do vaccine trial	1	0.18	47.13	
I don't really trust the vaccine is produced in Ghana	1	0.18	47.31	
I don't trust the vaccines	1	0.18	47.49	
I don't trust the white men	1	0.18	47.67	
I don't want any introduced disease in my system	1	0.18	47.85	
I don't want to die	1	0.18	48.03	
I have a funny feeling about some vaccines	1	0.18	48.21	
I have heard that the vaccine is not good	1	0.18	48.39	
I have heard that the vaccines are not good	1	0.18	48.57	
I have no expertise on it	1	0.18	48.75	
I have no symptoms of it	1	0.18	48.92	
I have not heard the world health organization have not come out with any vaccine, so i will not try any vaccine. It may be that it will not work.	1	0.18	49.10	
I have not tested positive	1	0.18	49.28	
I just delivered	1	0.18	49.46	
I just don't to do it	1	0.18	49.64	
I just won't	1	0.18	49.82	
I may be infected through the vaccine.	1	0.18	50.00	
I may die	1	0.18	50.18	
I may have some complications	1	0.18	50.36	
I may not know the effect of the vaccine.	1	0.18	50.54	
I may not know whether the vaccine is truly for Covid or not	1	0.18	50.72	
I might be infected rather	1	0.18	50.90	
I might get the infection	1	0.18	51.08	
I obey rules and regulations	1	0.18	51.25	
I think it's too risky	1	0.18	51.43	
I trust the process	1	0.18	51.61	
I will never try it because I don't believe them	1	0.18	51.79	
I will not like to be used at a lab rat	1	0.18	51.97	
I will not subject myself for experiment	1	0.18	52.15	
I will not use myself for a trial	1	0.18	52.33	
I will use the vaccine if its approved but not for trial	1	0.18	52.51	
I will wait for the vaccine.	1	0.18	52.69	
I won't	1	0.18	52.87	
I won't allow myself to be used as a lab rat	1	0.18	53.05	
I won't be Guinea pig	1	0.18	53.23	
I won't know the outcome of the vaccine so therefore I can't do it	1	0.18	53.41	
I would to see results in worse hit countries first before trying that.	1	0.18	53.58	
I wouldn't trust the source of the vaccine	1	0.18	53.76	
I wouldn't know the outcome	1	0.18	53.94	
I wouldn't want to risk	1	0.18	54.12	
I'll wait till it works and take it	1	0.18	54.30	
I'm afraid	3	0.54	54.84	
I'm more than convinced that the vaccine would not be made in Ghana but would be made by the Whites and I don't trust them	1	0.18	55.02	
I'm not a specimen	1	0.18	55.20	
I'm not affected by the virus	1	0.18	55.38	
I'm not ready to take any vaccine	1	0.18	55.56	
I'm not sick	1	0.18	55.73	
I'm not sure of side effects	1	0.18	55.91	
I'm not sure of its safety looking at the rush with which things are being done.	1	0.18	56.09	
I'm scared	3	0.54	56.63	
I'm scared	2	0.36	56.99	
I'm undecided.	1	0.18	57.17	
I'm very sure that I'm not infected	1	0.18	57.35	
I'm scared	1	0.18	57.53	
If vaccine is not discovered in Africa	1	0.18	57.71	
If yes, I have to get paid	1	0.18	57.89	
I'm not interested	1	0.18	58.06	
Insecurity to the human body	1	0.18	58.24	
Is new	1	0.18	58.42	
Is not good	1	0.18	58.60	
It can kill you	1	0.18	58.78	
It could be detrimental to my health	1	0.18	58.96	
It is spiritual hence no need for vaccine.	1	0.18	59.14	
It is a fraud. What is the source of the virus? What is in the vaccine?	1	0.18	59.32	
It is not approved by MoH	1	0.18	59.50	
It is not good	1	0.18	59.68	
It is too generous	1	0.18	59.86	
It is very dangerous	1	0.18	60.04	
It may be dangerous because its trial.	1	0.18	60.22	
It may kill me	1	0.18	60.39	
It might cause damages	1	0.18	60.57	
It might give other reactions that i might not know about	1	0.18	60.75	
It should be tested on the animals before	1	0.18	60.93	
It should be tested on animals first.	1	0.18	61.11	
It should be tried and tested, researched to be sure that it is safe before trying to test on us	1	0.18	61.29	
It should be tried on animals before humans	1	0.18	61.47	
It should be tried on animals first	1	0.18	61.65	
It should be tried where the virus originated area.	1	0.18	61.83	
It should try on animals first if it works before humans	1	0.18	62.01	
Its composition is not favorable	1	0.18	62.19	
It's my decision not to	1	0.18	62.37	
It's risky	1	0.18	62.54	
It's risky	1	0.18	62.72	
Just Scared of the outcome	1	0.18	62.90	
Just because it's a trial	1	0.18	63.08	
Just do not want to do it	1	0.18	63.26	
Just don't want	1	0.18	63.44	
Just don't want to be involved	1	0.18	63.62	
Just don't buy into that idea	1	0.18	63.80	
Just not ready	1	0.18	63.98	
Just will not do it	1	0.18	64.16	
Killing	1	0.18	64.34	
Let it be tried on citizens who produced the vaccines first before being use in Africa.	1	0.18	64.52	
Masks are more likely to save lives than vaccines	1	0.18	64.70	
May not be safe to use	1	0.18	64.87	
Might have another health implications on me	1	0.18	65.05	
Might not know outcome	1	0.18	65.23	
My age shows l have a week system. It should be tried on those with strong immune system	1	0.18	65.41	
My immune system is strong	1	0.18	65.59	
My immune system is strong	1	0.18	65.77	
N/A	4	0.72	66.49	
N/a	1	0.18	66.67	
Not Certain about the Vaccine	1	0.18	66.85	
No	2	0.36	67.20	
No	1	0.18	67.38	
No because I've not been tested of the virus	1	0.18	67.56	
No because officers should be sure that the vaccine works 100% and shouldn't have the intention of giving it a try on people. What if it causes death?	1	0.18	67.74	
No comfortable with that	1	0.18	67.92	
No experience in that field	1	0.18	68.10	
No I don't want to put my health on the line	1	0.18	68.28	
No idea	1	0.18	68.46	
No interested	1	0.18	68.64	
No particular reason	1	0.18	68.82	
No reason	3	0.54	69.35	
No reason	2	0.36	69.71	
No reason but won't participate	7	1.25	70.97	
No vaccine	2	0.36	71.33	
Not comfortable	2	0.36	71.68	
Not comfortable	1	0.18	71.86	
Not comfortable since I have very little Scientific background to understand enough.	1	0.18	72.04	
Not convinced about long term side effects of the vaccines	1	0.18	72.22	
Not having covid-19 sickness	1	0.18	72.40	
Not having the virus	1	0.18	72.58	
Not having time	1	0.18	72.76	
Not interested	16	2.87	75.63	
Not interested	1	0.18	75.81	
Not interested	1	0.18	75.99	
Not necessary	2	0.36	76.34	
Not necessary	1	0.18	76.52	
Not needed	1	0.18	76.70	
Not only vaccine can be used to treat Covid-19	1	0.18	76.88	
Not prepared	1	0.18	77.06	
Not ready	2	0.36	77.42	
Not safe	2	0.36	77.78	
Not sure	2	0.36	78.14	
Not sure I am in the mental state to do it.	1	0.18	78.32	
Not sure if it works	1	0.18	78.49	
Not sure of it	2	0.36	78.85	
Not sure of its effectiveness	1	0.18	79.03	
Not sure of potency of vaccine	1	0.18	79.21	
Not sure of side effects	4	0.72	79.93	
Not sure of the content	1	0.18	80.11	
Not sure of the effect of the vaccine	1	0.18	80.29	
Not sure of the side effects	1	0.18	80.47	
Not sure of the vaccine	5	0.90	81.36	
Not sure why	1	0.18	81.54	
Not too sure of vaccine efficacy and originality	1	0.18	81.72	
Nothing	11	1.97	83.69	
Nothing	5	0.90	84.59	
Noting	2	0.36	84.95	
People will stigma me	1	0.18	85.13	
Per the Information I have received	1	0.18	85.30	
Personal	2	0.36	85.66	
Personal	1	0.18	85.84	
Personal reasons	1	0.18	86.02	
Personal reasons	1	0.18	86.20	
Piloting comes with its own anomalies and I don't want to be a victim to such	1	0.18	86.38	
Politicians should be tried first	1	0.18	86.56	
Prefer not to disclose	3	0.54	87.10	
Safety reasons	1	0.18	87.28	
Scarce of its negative effect	1	0.18	87.46	
Scared	6	1.08	88.53	
Scared	1	0.18	88.71	
Scared of the vaccine	1	0.18	88.89	
Simply No	1	0.18	89.07	
Since I'm not infected	1	0.18	89.25	
Source Media was giving us information about the vaccine Should not take ok	1	0.18	89.43	
Still afraid of the vaccine	1	0.18	89.61	
Still new drug	1	0.18	89.78	
The chance of failure being high, I won't risk	1	0.18	89.96	
The risk involved	1	0.18	90.14	
The trial should start in the western world they should not come and try it in Africa	1	0.18	90.32	
The uncertainty of the outcome	1	0.18	90.50	
There is no disease	1	0.18	90.68	
There is no need	1	0.18	90.86	
There is no need since animals are available	1	0.18	91.04	
There is no vaccine	1	0.18	91.22	
There is no vaccine that can cure this virus	1	0.18	91.40	
They should openly inject animals with the one all nation tv station with the citizens been informed on the day of action. That the level at which we trust foreign vaccines to our own seem unsatisfactory to me.	1	0.18	91.58	
This is because I don't have the virus	1	0.18	91.76	
This is because, it's a trial vaccine	1	0.18	91.94	
Those who invent the vaccine should try it first.	1	0.18	92.11	
Too much scare surrounding it	1	0.18	92.29	
Uncertainty of the vaccine	1	0.18	92.47	
Undecided	2	0.36	92.83	
Unless it been approved, confirmed and satisfying by all stakeholders	1	0.18	93.01	
Unless the experiment takes like 2 years before for me to be sure.	1	0.18	93.19	
Unsafe for me	1	0.18	93.37	
Unwillingness to participate	1	0.18	93.55	
Vaccines should try with animals.  But not human beings.	1	0.18	93.73	
Vaccines to be tested sometimes have negative implications in the future.	1	0.18	93.91	
Very busy	1	0.18	94.09	
Very scared	1	0.18	94.27	
Wait till approved	1	0.18	94.44	
Want it to be properly tested before	1	0.18	94.62	
What if they try on me n it doesn't work, so will wait till it's done on someone before	1	0.18	94.80	
Whichever country that finds it should try it on her people. Africa is tired of these vaccine trials.	1	0.18	94.98	
Why me?	1	0.18	95.16	
Why should i put my life in such a risk	1	0.18	95.34	
Will not want to stand a trial-and-error chance	1	0.18	95.52	
Won't be a tested	1	0.18	95.70	
Wouldn't want to be used as a Guinea pig for any experiment.	1	0.18	95.88	
Afraid	1	0.18	96.06	
am not infected	1	0.18	96.24	
Authenticity	1	0.18	96.42	
because I fear	1	0.18	96.59	
because of potential risk of side effect	1	0.18	96.77	
don't want to take any risk	1	0.18	96.95	
fear contracting cvid-19 from vaccine	1	0.18	97.13	
I can't be used as someone's experiment.	1	0.18	97.31	
Don't feel it's safe	1	0.18	97.49	
I don't trust it, taking into consideration the various news making the airwaves	1	0.18	97.67	
i have never tested positive hence would not want to be used for a trial	1	0.18	97.85	
if it is not specified to be the solution, I can't be used for a trial but those who produced the so-called vaccine should be the first people to be used for the vaccine trial.	1	0.18	98.03	
Just don't want to	1	0.18	98.21	
lack of trust	1	0.18	98.39	
leadership by example	1	0.18	98.57	
Personal	1	0.18	98.75	
psychological effect	1	0.18	98.92	
Scared	1	0.18	99.10	
trials should be conducted in countries with highest infection rate	1	0.18	99.28	
Uncertain	1	0.18	99.46	
unless there has been a clinical trial in 3 phases	1	0.18	99.64	
vaccine testing is unsafe on humans	1	0.18	99.82	
what if it will kill me or has negative impact	1	0.18	100.00	
Total	558	100.00		
	


Table 2: Verbatim reasons cited for unwillingness to take COVID-19 vaccine 
If no why wouldn't accept to be immunized with a COVID-19 vaccine?	Freq.	Percent	Cum.	
Afraid	7	2.40	2.40	
Afraid	1	0.34	2.74	
Already stated	1	0.34	3.08	
Am afraid	1	0.34	3.42	
Am afraid	2	0.68	4.11	
Am afraid I will have side effects	1	0.34	4.45	
Am not affected	1	0.34	4.79	
Am not comfortable with it	1	0.34	5.14	
Am not sure of the vaccine	1	0.34	5.48	
Am okay	1	0.34	5.82	
Am scared	1	0.34	6.16	
Am scared	1	0.34	6.51	
Am sure of myself that I don't have the virus	1	0.34	6.85	
Because I am not entirely sure if it works	1	0.34	7.19	
Because I am not sick	1	0.34	7.53	
Because I don't have Corona	1	0.34	7.88	
Because I don't know the side effect	1	0.34	8.22	
Because I want to stick to the natural herbs	1	0.34	8.56	
Because I don't want to die	1	0.34	8.90	
Because it categorization it's not know	1	0.34	9.25	
Because it is not safe	1	0.34	9.59	
Because it is too deadly	6	2.05	11.64	
Because I don't have any education on the vaccine	1	0.34	11.99	
Because of the fear of fake vaccine	1	0.34	12.33	
Because there is no approved scientific drugs for it.	1	0.34	12.67	
Because there is no approved vaccine yet	1	0.34	13.01	
Because there's no vaccine yet and I wouldn't allow myself being injected with a wrong vaccine which will bring other complications. Unless we all hear of a real vaccine, I wouldn't go through the vaccination process	1	0.34	13.36	
Can't be trusted	1	0.34	13.70	
Can't risk my life	1	0.34	14.04	
Confidential please	1	0.34	14.38	
Course damage to his or her health	1	0.34	14.73	
Dangerous	1	0.34	15.07	
Do not trust the vaccine	1	0.34	15.41	
Do we have it	1	0.34	15.75	
Don't know whether it will work	1	0.34	16.10	
Don't like the vaccine	1	0.34	16.44	
Don't want them to be used for an experiment	1	0.34	16.78	
Don't the outcome	1	0.34	17.12	
Don't trust the whites	1	0.34	17.47	
Even the country that came out with the vaccine were even dying more.	1	0.34	17.81	
Fear reason	1	0.34	18.15	
Fear	4	1.37	19.52	
Fear of side effects	1	0.34	19.86	
Fear the western world	1	0.34	20.21	
For fear of side effect	1	0.34	20.55	
For my own safety	1	0.34	20.89	
Have gained immunity	1	0.34	21.23	
How would you know if they are infecting you with different virus	1	0.34	21.58	
I don't want to die	1	0.34	21.92	
I am afraid	2	0.68	22.60	
I am afraid of future serious effects of the vaccine	1	0.34	22.95	
I am not sick	2	0.68	23.63	
I am not sure of the safety of the vaccine	1	0.34	23.97	
I cannot trust the white man on this vaccine trial	1	0.34	24.32	
I can't risk my life	1	0.34	24.66	
I can't trust vaccines	1	0.34	25.00	
I don't believe in taking vaccine	1	0.34	25.34	
I don't believe it	1	0.34	25.68	
I don't believe on the vaccine because no scientists came out with the vaccine	1	0.34	26.03	
I don't believe that's safe	1	0.34	26.37	
I don't have the disease	1	0.34	26.71	
I don't have the disease	1	0.34	27.05	
I don't know if it's safe because there were so many rumors about it	1	0.34	27.40	
I don't know the side effects after the immunization	1	0.34	27.74	
I don't know the source of it	1	0.34	28.08	
I don't like	1	0.34	28.42	
I don't need it	1	0.34	28.77	
I don't take injection	1	0.34	29.11	
I don't think it's needed	1	0.34	29.45	
I don't trust any vaccine	1	0.34	29.79	
I don't trust any vaccine work up to 100%	1	0.34	30.14	
I don't trust anyone	2	0.68	30.82	
I don't trust it	1	0.34	31.16	
I don't trust our leaders	1	0.34	31.51	
I don't trust the process	1	0.34	31.85	
I don't trust the source	1	0.34	32.19	
I don't trust the vaccine	1	0.34	32.53	
I don't trust the vaccine	1	0.34	32.88	
I don't trust the western world	1	0.34	33.22	
I don't trust the white	1	0.34	33.56	
I don't trust where it's coming from.	1	0.34	33.90	
I don't want it	1	0.34	34.25	
I don't want to die	3	1.03	35.27	
I don't want to take any medicine of COVID-19	1	0.34	35.62	
I don't want to take chances	1	0.34	35.96	
I don't buy the idea	1	0.34	36.30	
I don't trust some vaccines	1	0.34	36.64	
I don't trust the vaccines	1	0.34	36.99	
I don't want any injection	1	0.34	37.33	
I don't want to try	1	0.34	37.67	
I have a strong immune system	1	0.34	38.01	
I have no interest in it	1	0.34	38.36	
I just don't want it	1	0.34	38.70	
I just don't want injection	1	0.34	39.04	
I still don't trust it	1	0.34	39.38	
I was not interested	1	0.34	39.73	
I will allow if I test positive and the vaccine is approved	1	0.34	40.07	
I will not do it	1	0.34	40.41	
I will not trust any vaccine for covid19	1	0.34	40.75	
I will only do that if it tested on animals and they don't die.	1	0.34	41.10	
I will rather boost my immune system	1	0.34	41.44	
I won't	2	0.68	42.12	
I would to see results in worse hit countries first	1	0.34	42.47	
I'm afraid	1	0.34	42.81	
I'm not Interested	1	0.34	43.15	
I'm not infected by the virus	1	0.34	43.49	
I'm ready to take any vaccine	1	0.34	43.84	
I'm scared to die	1	0.34	44.18	
I'm still wouldn't be sure of its safety so I will wait and see.	1	0.34	44.52	
I've not tested positive	1	0.34	44.86	
If I have not seen what is satisfactory to me	1	0.34	45.21	
If I don't have it why be vaccinated	1	0.34	45.55	
If only it's from Ghanaian doctor but not prescribed by foreign doctors.	1	0.34	45.89	
I'm not sick	1	0.34	46.23	
Insecurity to the human body	1	0.34	46.58	
Is a New vaccine	1	0.34	46.92	
Is it safe?	1	0.34	47.26	
Is not good	1	0.34	47.60	
It is a fraud	1	0.34	47.95	
It is not safe	1	0.34	48.29	
It may compact my immune system and rather infect me	1	0.34	48.63	
It may give pains	1	0.34	48.97	
It may kill	1	0.34	49.32	
It might be dangerous	1	0.34	49.66	
It my immune system is strong	1	0.34	50.00	
It will be dangerous for the modern world we now live.	1	0.34	50.34	
It will bring out other sickness	1	0.34	50.68	
It's a trial vaccine	1	0.34	51.03	
It's fatal	1	0.34	51.37	
It's foolish	1	0.34	51.71	
It's risky	1	0.34	52.05	
Just don't know why?	1	0.34	52.40	
Let it be tried on citizens who produced the vaccines first before being use in Africa.	1	0.34	52.74	
Masks are more likely to save lives than vaccines	1	0.34	53.08	
Maybe I'll die	1	0.34	53.42	
Money first	1	0.34	53.77	
My immune system is strong	1	0.34	54.11	
My safety	1	0.34	54.45	
N/A	4	1.37	55.82	
Not Certain of Vaccine	1	0.34	56.16	
Needs to take some time to know the after effects some years to come	1	0.34	56.51	
No	4	1.37	57.88	
No apparent reason.	1	0.34	58.22	
Not approved by MoH	1	0.34	58.56	
No because I don't know the side effects.	1	0.34	58.90	
No disease	1	0.34	59.25	
No explanations	1	0.34	59.59	
No idea	1	0.34	59.93	
No in my time	1	0.34	60.27	
No need	2	0.68	60.96	
No need	1	0.34	61.30	
No reason	2	0.68	61.99	
No reason	2	0.68	62.67	
No trust	1	0.34	63.01	
No trust	1	0.34	63.36	
No unless it has been tried, tested and approved by health directorate	1	0.34	63.70	
No vaccine can cure	1	0.34	64.04	
Not certain about the immunization	1	0.34	64.38	
Not certain if it's authentic	1	0.34	64.73	
Not convinced	2	0.68	65.41	
Not good	2	0.68	66.10	
Not important	1	0.34	66.44	
Not interested	1	0.34	66.78	
Not interested	9	3.08	69.86	
Not interested	1	0.34	70.21	
Not necessary	2	0.68	70.89	
Not necessary	1	0.34	71.23	
Not needed	1	0.34	71.58	
Not now unless I'm sure of the drug	1	0.34	71.92	
Not now, it should be tried and tested before	1	0.34	72.26	
Not safe	1	0.34	72.60	
Not safe	1	0.34	72.95	
Not sick of Covid-19	1	0.34	73.29	
Not sick of covid-19	1	0.34	73.63	
Not sure about the side effects of the vaccine	1	0.34	73.97	
Not sure of it	1	0.34	74.32	
Not sure of its effectiveness	1	0.34	74.66	
Not sure of the vaccine	1	0.34	75.00	
Not sure of vaccine	1	0.34	75.34	
Not tested	1	0.34	75.68	
Not until it has been duly authorized by recommended sources	1	0.34	76.03	
Not until its W.H.O approved	1	0.34	76.37	
Nothing	4	1.37	77.74	
Nothing	1	0.34	78.08	
Nothing really	1	0.34	78.42	
Noting	2	0.68	79.11	
People are you will die if you take it	1	0.34	79.45	
People do say that immunization is harmful.	1	0.34	79.79	
Personal	1	0.34	80.14	
Personal	3	1.03	81.16	
Personal reasons	1	0.34	81.51	
Personal reasons	1	0.34	81.85	
Religious reasons	1	0.34	82.19	
Risky	1	0.34	82.53	
Safety issues	1	0.34	82.88	
Same	1	0.34	83.22	
Scared	4	1.37	84.59	
Scared of it	1	0.34	84.93	
Scared to die	2	0.68	85.62	
Simply No	1	0.34	85.96	
The things are not working	1	0.34	86.30	
The vaccine is not good	1	0.34	86.64	
There is no vaccine known	1	0.34	86.99	
There is no recommended approved vaccine.	1	0.34	87.33	
They said its poisonous from the white people	1	0.34	87.67	
They should use it on someone first before	1	0.34	88.01	
They should use it first	1	0.34	88.36	
Think the immune boosters are helping	1	0.34	88.70	
To avoid any side effect	1	0.34	89.04	
Too much bad news about the vaccine	1	0.34	89.38	
Too much scare surrounding it	1	0.34	89.73	
Too soon	1	0.34	90.07	
Uncertainty of the vaccine	1	0.34	90.41	
Undecided	1	0.34	90.75	
Unless confirmed by our recognized Health Ministries	1	0.34	91.10	
Unless it has been tested and proven to have any or major side effects; I'd rely on the proper handwashing, vitamins intake, etc. (COVID-19 preventive measures).	1	0.34	91.44	
Unless it is widely accepted worldwide	1	0.34	91.78	
Unless the whole world is partaking	1	0.34	92.12	
Unless, established from WHO	1	0.34	92.47	
Until I see it work from where it started	1	0.34	92.81	
Until a prove of it is provided.	1	0.34	93.15	
Until proven to not have an effect on my health (asthma and lung issues)	1	0.34	93.49	
Vaccines take longer years to know side effects	1	0.34	93.84	
Which country develops the vaccine	1	0.34	94.18	
Why would I?	1	0.34	94.52	
Will accept it, only when its widely accepted world wide	1	0.34	94.86	
Would only accept if it is recognized globally and safe to use	1	0.34	95.21	
Am not infected	1	0.34	95.55	
I can't tell	1	0.34	95.89	
I don't like vaccine	1	0.34	96.23	
I don't trust them	1	0.34	96.58	
I fear	1	0.34	96.92	
Just don't want to be immunized	1	0.34	97.26	
May be yes may be no	1	0.34	97.60	
No reason	1	0.34	97.95	
Not interested	1	0.34	98.29	
Nothing	1	0.34	98.63	
Nothing	1	0.34	98.97	
Uncertain	1	0.34	99.32	
Until I see that those who have been immunized have no Effects i will not	1	0.34	99.66	
Will only accept it after I hear its actually working	1	0.34	100.00	
Total	292	100.00		
	
